# Right Superior Frontal Gyrus Cortical Thickness in Pediatric ADHD

**DOI:** 10.1177/10870547221110918

**Published:** 2022-07-11

**Authors:** Tasmia Hai, Rose Swansburg, Cynthia K. Kahl, Hannah Frank, Kayla Stone, Jean-François Lemay, Frank P. MacMaster

**Affiliations:** 1 University of Calgary, AB, Canada; 2 University of Alberta, Edmonton, Canada; 3 Mount Royal University, Calgary, AB, Canada; 4 Addictions and Mental Health Strategic Clinical Network, Calgary, AB, Canada

**Keywords:** ADHD, cortical thickness, superior frontal gyrus, executive functions

## Abstract

**Objective:**

We investigated the right Superior Frontal Gyrus (right-SFG) and Anterior Cingulate Cortex (ACC) in children with ADHD and their clinical relevance with Executive Function (EF) and ADHD symptom severity.

**Methods:**

About 26 children with ADHD and 24 typically developing children (TDC; 7‒16 years) underwent Magnetic Resonance Imaging (MRI) and completed an EF assessment battery.

**Results:**

Significantly thinner right-SFG in the ADHD group was found compared to the TDC group (*t* (48) = 2.81, *p* = .007, Cohen’s *d* = 0.84). Linear regression models showed that 12.5% of inattention, 13.6% of hyperactivity, and 9.0% of EF variance was accounted for by the right-SFG thickness.

**Conclusions:**

Differences in the right-SFG thickness were found in our ADHD group and were associated with parent ratings of inattentive and hyperactive symptoms as well with EF ratings. These results replicate previous findings of thinner right-SFG and are consistent with the delayed cortical maturation theory of ADHD.

ADHD is a prevalent neurodevelopmental disorder with estimates of 5% to 9% in Canadian school-aged children (Brault & Lacourse, 2012; Polanczyk et al., 2014). Typically, ADHD symptoms include inattention or overactivity and impulsivity that are developmentally inappropriate (American Psychiatric Association, 2013). While ADHD is considered a childhood disorder, ADHD symptoms often persist into adolescence and adulthood (Biederman et al., 2010; Wilens & Spencer, 2010).

Symptoms of ADHD affect development across multiple domains including academic (Wolraich, 2005), social (Hoza et al., 2005), and motor functioning (Kaiser et al., 2015), and when left untreated, can develop other mental health disorders such as anxiety and depression (Furczyk & Thome, 2014). Moreover, studies have reported adverse long-term consequences such as developing conduct disorder, substance use disorder, increased suicidal thoughts, and increased mortality rate in people diagnosed with ADHD (Dalsgaard et al., 2015; Dunne et al., 2014; Furczyk & Thome, 2014; Rowe et al., 2010). A recent estimate of the financial and socio-economic burden from the United States suggested that the direct economic cost of raising a child with ADHD is about five times higher than raising a child without ADHD (Zhao et al., 2019).

Children with ADHD often exhibit significant Executive Function (EF) challenges, an umbrella term used to describe higher-order, goal-oriented processes. These EF skills typically include planning, inhibition, and working memory (Biederman et al., 2004; Huang-Pollock et al., 2012; Toplak et al., 2005; Willcutt et al., 2005). EF skills play important roles in an individual’s ability to regulate their thoughts, actions, emotions, and behaviors. They are also important for academic performance, such as reading, writing, and mathematics (Cortés Pascual et al., 2019). While numerous studies have investigated EF challenges in children with ADHD, the findings are inconsistent with studies showing variable EF performance across the different domains (Huang-Pollock et al., 2012; Willcutt et al., 2005). Furthermore, comorbidities and intellectual functioning can impact EF performance (Bental & Tirosh, 2007; Kofler et al., 2019).

Diagnosis of ADHD (and other disorders) are frequently made based on the Diagnostic and Statistical Manual of Mental Disorders-Fifth Edition (DSM-V) symptom criteria (American Psychiatric Association, 2013). These symptom clusters are based on behavior reported by parents, teachers, and clinicians with limited emphasis on biological relevance (Pallanti & Salerno, 2020). To improve diagnosis and treatment outcomes, researchers are increasingly interested to investigate potential biomarkers of different psychiatric and neurodevelopmental disorders.

Given the heterogeneous presentation of ADHD symptoms, multiple theoretical models with different biomarkers have been proposed. For example, the dopamine transporter genes (DAT1, norepinephrine transporter genes [NET1]), and neuropsychological endophenotypes (Barkley et al., 2001; Castellanos et al., 2006; Faraone et al., 2014; Franke et al., 2018) are some of the potential candidate biomarkers. In the last few years, cortical thickness has emerged as one promising neuroimaging biomarker (Cortese & Coghill, 2018; Hoogman et al., 2019). Cortical thickness is a measure of cortical columnar structure, which generally suggests changes in cellular maturation in the cortex due to dendritic arborization, pruning, and myelination (Fischl & Dale, 2000). Cortical thickness has been of interest for studying both normal development as well as the potential risk factors of psychiatric disorders (Fischl & Dale, 2000). Previous cross-sectional studies conducted in individuals with ADHD have reported reduced cortical thickness in the Frontal-Striatal Pathway (FSP), specifically in the Anterior Cingulate Cortex (ACC) and Superior Frontal Gyrus (SFG; Bledsoe et al., 2013; Shaw et al., 2007; Yang et al., 2015). A recent mega-analysis conducted by the Enigma Consortium with over 2,200 ADHD participants between the ages of 4 and 62 years old (*M*_age_ = 19.22 years) found lower surface areas in the frontal, cingulate, and temporal regions and lower cortical thickness in the fusiform gyrus and temporal pole (Hoogman et al., 2019).

While neuroanatomical differences in ADHD have been commonly studied, a limited number of studies to date have investigated the dimensional relationship between EF, behavioral symptoms, and cortical thickness in the SFG and ACC to further support the clinical relevance of these anatomical regions (Bledsoe et al., 2013). Given that previous functional MRI studies have identified both hypoactivated and hyperactivated areas during attention and inhibition tasks in the right prefrontal cortex regions, basal ganglia, cerebellum, anterior cingulate cortex, and supplementary motor area (Cortese & Coghill, 2018; Cortese et al., 2014; Rubia, 2018), it is possible that cortical thickness of SFG and ACC may be related to neuropsychological performance-based tasks related to inhibition and working memory.

With recent criticisms surrounding the categorical understanding of different mental health disorders, there has been an increased interest and need to study symptoms along a continuum as suggested by the Research Domain Criteria (RDoC; Kraemer, 2007). Additionally, there are inconsistencies in the present literature regarding different neuroanatomical correlates of ADHD. These inconsistencies could be due to the heterogeneity of the ADHD sample size, diagnostic criteria, treatment effects, improved MRI resolution, and/or comorbid disorders. As a result, replicating previous findings is essential to increase our understanding of the neuroanatomical correlates of ADHD to provide targeted treatment options.

## Current Study

The purpose of the current study was two-fold: the primary goal was to investigate the neuroanatomical biomarkers of ADHD, in particular cortical thickness in the right-SFG and ACC regions in order to replicate previous findings ((Yang et al., 2015) in a non-medication naïve sample of ADHD. Secondly, we investigated the clinical relevance of the right-SFG and ACC cortical thickness with ADHD core symptoms of inattention and hyperactivity and EF performance on response inhibition and working memory tasks and parent ratings of EF using a dimensional perspective according to the RDoC (Casey et al., 2014). Behavior and EF tasks were selected as some of the main challenges observed by parents and teachers in children with ADHD.

We expected thinner cortical thickness in the SFG and ACC in children with ADHD compared to typically developing children (TDC). Even though previous research studies have reported abnormalities in both left and right frontal cortices, some studies have proposed that the right hemisphere is predominantly altered in children with ADHD (Rubia et al., 1999, 2010). As a result, the current study expected to observe abnormalities in the right frontal regions. We also expected to observe correlations between performance on EF tasks, parent ratings, and cortical thickness in ACC and right-SFG. Given the limited research investigating cortical thickness and EF performance, we did not have a priori hypothesis about the direction of the correlational relationship.

## Materials and Methods

### Participants

Children between the ages of 7 and 16 years old, with a confirmed diagnosis of ADHD and without a diagnosis of ADHD, were recruited from Calgary region pediatric clinics, the general community, and social media, including Facebook and Twitter. The study received research ethics approval from the Conjoint Health Research Ethics Board (CHREB) at the University of Calgary (REB19-0499). All parents provided consent for participation and the children provided assent.

### Inclusion Criteria

Participants in the ADHD group had to have (1) a confirmed ADHD diagnosis from a healthcare professional, verified by an experienced Developmental Pediatrician (author JFL), (2) a behavior rating score greater than 65 (T-score) on the Conners-3 parent rating scale, (3) confirmation of ADHD diagnosis on the Mini-International Neuropsychiatric Interview for Children and Adolescents (MINI-KID; Sheehan et al., 2010), and (4) no intellectual disability (a cognitive screener standard score >80). All participants in the ADHD group underwent a 48-hour washout period in order to decrease the impact of stimulant medications on their performance. They were allowed to continue taking their other prescribed medication such as anti-depressants. Participants in the TDC group did not have any psychiatric diagnoses, including ADHD. This was confirmed through the structured clinical interview, MINI-KID.

### Exclusion Criteria

Participants were ineligible to take part in the study if they had (1) a diagnosis of Autism Spectrum Disorder (ASD), traumatic brain injury, seizure disorder, intellectual disability, or any other medical conditions that could impact their cognitive scores, (2) metal in their body that would prevent them from taking part in MRI, and (3) unable to complete a 48-hour medication washout period.

### Executive Function Measures

Participants from both groups completed neuropsychological performance-based assessments related to inhibition and working memory. Specifically, for measuring both visual and verbal working memory, the Digit Span Backwards and Spatial Span Backwards subtests from the Wechsler Intelligence Scale for Children-Fifth Edition (WISC-V) and WISC-V Integrated (Wechsler & Kaplan, 2015) were used. Conners-3 Continuous Performance Test, third Edition (CPT III) was used to measure response inhibition (Conners, 2014). Psychometric properties of both working memory and response inhibition tasks suggested good reliability and validity (Conners, 2014; Na & Burns, 2016; Watkins et al., 2018). Parents also completed rating scales using the Behavior Rating Inventory of Executive Function (BRIEF-2) to report on executive dysfunction.

### MRI Acquisition Protocol

All participants underwent a high-resolution MRI T1-weighted sequence using a 3 Tesla General Electric Discovery 750W MRI scan with a 32-channel head coil. Structural MRI parameters were as follows: TR = 8.2 ms, TE = 3.2 ms, flip angle = 10°, field of view (FOV) = 256 mm^2^, acquisition matrix size = 300 × 300, and voxels = 0.8 mm^3^ isotropic.

### Cortical Thickness Analysis

FreeSurfer 6.0 was used for cortical surface reconstruction (Fischl & Dale, 2000). FreeSurfer is a set of tools that construct models of the boundary between white matter (WM) and cortical gray matter (GM). The pipeline consists of several stages and includes motion correction, removal of non-brain matter such as skull and dura matter, an algorithm for finding and correcting the topological defects in the initial WM/GM surface, a method to deform the mesh for reconstructing the inner and pial surfaces, automated Talairach transformation, subcortical white and gray matter structures, and surface deformation for optimal differentiation of white and gray matter and gray and cerebrospinal fluid intensity boundaries. A detailed description of the Free Surfer processing is described online (https://surfer.nmr.mgh. harvard.edu/fswiki/FreeSurferMethodsCitation; Dale et al., 1999; Fischl & Dale, 2000). This measurement technique has been validated manually (Kuperberg et al., 2003).

### Statistical Analysis

All analyses were conducted using SPSS version 25. Data were inspected for missing values, outliers, normality, linearity, and homogeneity of variance to meet the assumptions for parametric analysis. Independent *t*-tests were conducted to measure group differences between the right and left superior frontal gyrus and caudal and rostral anterior cingulate cortex. Multivariate analysis of variance (MANOVA) was used to investigate group differences in EF performance. Pearson Correlations were conducted to investigate the relationships between cortical thickness, EF performance, and ADHD symptomology. Lastly, linear regressions were conducted to investigate the relationships between cortical thickness, EF assessments, and behavioral symptoms of ADHD for the combined sample (both ADHD and TDC groups). Benjamini-Hochberg principle was used to correct for multiple comparisons (Benjamini & Hochberg, 1995).

#### Post hoc analysis

Biological sex differences in the EF measures and cortical thickness were conducted using independent sample *t*-tests and MANOVA to investigate sex differences.

### Procedures

All the assessments and MRI scanning took place at the Alberta Children’s Hospital (ACH) between June 2019 and November 2019. Participants first completed screening measures to ensure eligibility. Parents of children with ADHD and the TDC group completed questionnaires separately from their child to indicate their perception of their child’s Inattention and Hyperactivity ratings. After completing screening measures (e.g., MINI-KID and the cognitive screener), participants completed additional neuropsychological measures (e.g., EF assessments). The study’s neuroimaging portion took about 75 minutes to complete, and participants could choose to have a caregiver sit beside them while in the scan. This scan time was chosen to ensure MRI sequences could be repeated if needed and to allow participants to take breaks. All participants were scanned by the same team of ACH diagnostic imaging staff on the same scanner with identical scanning parameters to ensure data acquisition consistency.

## Results

### Participant Characteristics

A total of 55 participants consented to the study. Two participants were excluded because they did not meet study eligibility criteria: one ASD diagnosis, one failure to observe the 48-hour medication washout period. Also, one participant withdrew within 1 hour of joining due to extreme shyness and anxiety. Lastly, two additional participants were excluded due to being outliers, as indicated through quality control measures completed on cortical thickness outcomes according to Enigma Consortium protocols (available freely for download from http://enigma.ini.usc.edu/protocols/imaging-protocols/).

A final sample of 26 children with ADHD (*M* = 11.61 years, *SD* = 2.5; *n* = 13 males) and 24 typically developing children (*M* = 10.89 years, *SD* = 2.5; *n* = 13 males) completed the study. There were no age or biological sex differences between groups. As expected, there were significant group differences in ADHD symptoms, as reported by parents on the Conners-3 rating scale. Specifically, parents of children with ADHD endorsed higher levels of Inattentive (*t* (48) = 7.71, *p* < .001, Cohen’s *d* = 1.96) and hyperactive/impulsivity (*t* (48) = 8.86, *p* < .001, Cohen’s *d* = 2.48) symptoms compared to the TDC group. There was also significant difference in performance on the WISC-V Arithmetic subtest (*t* (48) = 2.26, *p* = .029, Cohen’s *d* = 0.65). No other significant group differences in the intellectual functioning screener were observed: WISC-V Integrated Vocabulary subtest (*t* (48) = 1.18, *p* = .24, Cohen’s *d* = 0.33) and WISC-V Integrated Block Design subtest (*t* (48) = 0.46, *p* = .65, Cohen’s *d* = 0.13). Parent ratings of EF indicated significant EF difficulties across all three primary indices on the BRIEF-2, Behavior Regulation Index ((*t* (48) = 7.72, *p* < .001, Cohen’s *d* = 2.16), Emotion Regulation Index (*t* (48) = 5.42, *p* < .001, Cohen’s *d* = 1.52), and Cognitive Regulation Index (*t* (48) = 9.14, *p* < .001, Cohen’s *d* = 2.57). Lastly, there were no other significant differences in demographic information between the two groups (see Table 1 for demographic information).

**Table 1.** Participant Characteristic Information, Including Demographic Information, Intellectual Functioning Test Results, and ADHD Symptoms.

Variable	ADHD group (*n* = 26)		TDC (*n* = 24)		*t*	Cohen’s *d*	*p*-Value
	*M*	*SD*	*M*	*SD*			
Age (years)	11.61	2.53	10.89	2.54	1.00	0.28	.32
Conners-3 inattention (*t*-score)	75.69	11.40	54.25	7.74	7.83	2.20	<.001**
Conners-3 hyperactivity/impulsivity (*t*-score)	78.81	11.67	53.25	8.30	8.97	2.52	<.001**
WISC-V integrated vocabulary	103.08	12.73	107.3	12.51	1.18	0.33	.244
WISC-V integrated block design	106.53	11.98	108.13	12.66	0.46	0.13	.651
WISC-V arithmetic	96.54	13.47	103.75	8.75	2.26	0.653	.029*
BRIEF-2 Behavior Regulation Index (BRI)	67.62	10.89	48.88	5.64	7.72	2.16	<.001**
BRIEF-2 Emotion Regulation Index (ERI)	64.04	12.37	49.04	6.52	5.42	1.52	<.001**
BRIEF-2 Cognitive Regulation Index (CRI)	69.92	9.60	49.29	6.09	9.14	2.57	<.001**
Biological sex	*n*	%	*n*	%	χ^2^	*p* Value	
Male	13	50	13	50	0.02	.86	
Female	13	52	11	48			
Handedness							
Right	21	80.8	22	91.7	1.23	.27	
Left	5	19.2	2	8.3			
Medication							
Yes	22	84.6	1	4.2			
Methylphenidate	7	31.8					
Amphetamine	4	18.2					
Alpha-2 adrenergic agonist	1	4.5					
Antidepressant	2	9.1					
Combination of stimulant and non-stimulants	8	36.4					
Other (non-psychiatric)			1	4.2			
No	4	15.4	23	95.8			
Parent income							
Below Alberta median family income (under $99,000)	9	34.6	3	12.5	3.35	0.067	
Above Alberta median family income (above $99,000)	17	65.4	21	87.5			
Ethnicity							
Caucasians	23	88.5	15	62.5	8.42	0.077	
Asians	1	3.8	4	16.7			
First nations/metis	1	3.8	0	0			
Other	1	3.8	5	20.8			

*Note.* Alberta median income data was obtained from Statistics Canada (2018), Wechsler Intelligence Scale for Children-Fifth Edition.

* *p* < .05. **p < .01.

### Cortical Thickness

#### Group differences

**Figure 1.**
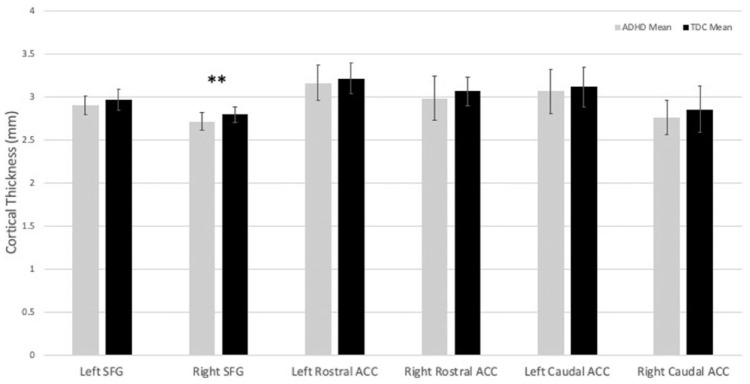
Cortical thickness of the right and left caudal anterior cingulate, superior frontal gyrus, and rostral anterior cingulate in children with ADHD (*n* = 26) and typically developing controls (TDC; *n* = 24) with error bars denoting the standard deviation. **p < .01.

**Figure 2.**
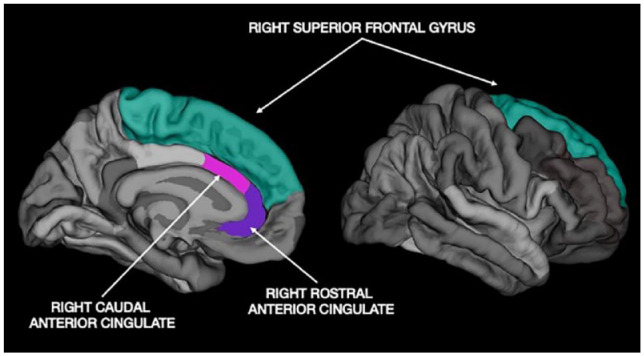
Parcellated image of the medial and lateral view of the right superior frontal gyrus (right-SFG), and medial view of right rostral and caudal anterior cingulate cortex (ACC) from free surfer 6.0.

Table 2 summarizes the cortical thickness findings. Independent *t*-tests indicated thinner cortex in the ADHD group compared to TDC in the right and left SFG: right-SFG (*t* (48) = 2.81, *p* = .007, Cohen’s *d* = 0.84) and left SFG (*t* (48) = 1.93, *p* = .05, Cohen’s *d* = 0.52). No significant group differences in cortical thickness were observed in the right or left rostral and caudal ACC (Figures 1 and 2). After correcting for multiple comparisons using the Benjamini-Hochberg principle (Benjamini & Hochberg, 1995), only the right-SFG results were statistically significant.

**Table 2.** Cortical Thickness Measurements of the ADHD and Typically Developing Control (TDC) Groups.

	ADHD (*n* = 26)		TDC (*n* = 24)		*t*	*p*-Value	Cohen’s *d*
	*M*	*SD*	*M*	*SD*			
Right hemisphere							
Superior frontal gyrus	2.71	0.10	2.79	0.09	−2.81	.007	0.84**
Rostral anterior cingulate cortex	2.98	0.26	3.06	0.17	−1.24	.222	0.36
Caudal anterior cingulate cortex	2.76	0.20	2.85	0.27	1.32	.195	0.38
Left hemisphere							
Superior frontal gyrus	2.90	0.11	2.96	0.12	−1.98	.054	0.52
Rostral anterior cingulate cortex	3.16	0.21	3.21	0.18	−0.89	.380	0.26
Caudal anterior cingulate cortex	3.06	0.26	3.11	0.23	−0.76	.450	0.20

** *p* < .01.

#### Sex differences

Independent sample *t*-tests were conducted to investigate the difference in cortical thickness between males and females independent of group difference. Results indicated that significant biological sex difference existed for the cortical thickness of right-SFG (*t* (48) = 1.99, *p* = .05, Cohen’s *d* = 0.57) and right rostral ACC (*t* (48) = 2.40, *p* = .02, Cohen’s *d* = 0.69). However, after correcting for multiple comparisons using the Benjamini-Hochberg principle (Benjamini & Hochberg, 1995), these biological sex differences were no longer statistically significant.

#### Group by sex differences

Six different ANOVA were conducted to investigate the interaction effect of cortical thickness with sex and ADHD diagnosis. No significant interaction effect was observed in all six regions of interest.

### Executive Functions

EF assessment results are presented in Table 3. MANOVA did not show any group differences in EF performance between the ADHD and TDC groups (*F* (5, 44) = 1.41, *p* = .24, *Wilks Lambda* = .14). However, univariate analysis of variance showed that children with ADHD made more perseverative errors than the TDC group on the CPT-3 task (*F* (1, 48) = 5.29, *p* = .026, *Partial Eta Squared* = .10). No other significant differences in performance were observed. There was also no statistically significant biological sex difference in performance on the EF tasks (*F* (5, 44) = 0.94, *p* = .49, *Wilks Lambda* = .09).

**Table 3.** Executive Function Performance Scores of the ADHD and Typically Developing Control (TDC) Groups.

Variables	ADHD (*n* = 26)		TDC (*n* = 24)		*F*	*p*-Value	MANOVA
	*M*	*SD*	*M*	*SD*			Partial Eta squared
Working memory							
Digit span backwards	97.31	12.10	102.08	11.12	2.10	.15	0.04
Spatial span backwards	101.34	15.59	101.25	13.77	0.001	.98	0.00
Response inhibition							
CPT-3 omission errors	64.08	16.99	57.38	15.08	2.16	.15	0.04
CPT-3 commission errors	56.27	7.34	55.29	7.65	0.21	.65	0.04
CPT-3 perseverative errors	67.62	16.19	57.00	16.42	5.29	.026	0.10*

* *p* < .05.

### Brain-Behavior Relationships

#### Correlations

Table 4 summarizes the different correlations that were conducted.

**Table 4.** Correlations Between Executive Functions, ADHD Symptoms, and Right SFG Cortical Thickness for both ADHD and Typically Developing Control (TDC) Groups Combined.

		1	2	3	4	5	6	7	8	9	10	11
1	Right SFG cortical thickness	—	*r* = −211	*r* = .165	*r* = −.164	*r* = −.117	*r* = −.009	*r* = −.353*	*r* = −.369**	*r* = −.270	*r* = −.183	*r* = −.295*
			*p* = 141	*p* = 252	*p* = .254	*p* = .419	*p* = .950	*p* = .012	*p* = .008	*p* = .058	*p* = .204	*p* = .038
2	CPT-3: Omission		—	*r* = .161	*r* = .613**	*r* = −437**	*r* = −262	*r* = .418**	*r* = .325*	*r* = .242	*r* = −.038	*r* = .254
				*p* = .264	*p* = .000	*p* = .002	*p* = .066	*p* = .003	*p* = .021	*p* = .091	*p* = .794	*p* = .075
3	CPT-3: Commission			—	*r* = .467**	*r* = −.377**	*r* = −.474**	*r* = .000	*r* = .133	*r* = .188	*r* = .216	*r* = .167
					*p* = .001	*p* = .007	*p* = .001	*p* = .999	*p* = .355	*p* = .191	*p* = .131	*p* = .247
4	CPT-3: Perseveration				—	*r* = −.222	*r* = −.303*	*r* = .317*	*r* = .337*	*r* = .396**	*r* = .137	*r* = .332*
						*p* = .121	*p* = .033	*p* = .025	*p* = .017	*p* = .004	*p* = .342	*p* = .018
5	WISC-V spatial span					—	*r* = .229	*r* = −.034	*r* = −.115	*r* = −.084	*r* = .085	*r* = .001
							*p* = .110	*p* = .813	*p* = .427	*p* = .563	*p* = .559	*p* = .992
6	WISC-V digit span						—	*r* = −.047	*r* = −.233	*r* = −.235	*r* = −.165	*r* = −.149
								*p* = .747	*p* = .103	*p* = .101	*p* = .253	*p* = .302
7	Conners-inattention							—	*r* = .794**	*r* = .732**	*r* = .550**	*r* = .795**
									*p* = .000	*p* = .000	*p* = .000	*p* = .000
8	Conners-3 hyperactive/impulsivity								−	*r* = .865**	*r* = .567**	*r* = .779**
										*p* = .000	*p* = .000	*p* = .000
9	BRIEF-2 BRI									—	*r* = .741**	*r* = .834**
											*p* = .000	*p* = .000
10	BRIEF-2 ERI										—	*r* = 677**
												*p* = .000
11	BRIEF-2 CRI											—

* Correlation is significant at the .05 level (two-tailed).

** Correlation is significant at the .01 level (two-tailed).

##### Executive function performance measures

No significant correlations were observed between right-SFG cortical thickness and performance on the different EF tasks.

##### Executive function parent ratings

Significant negative correlation was observed between right-SFG and BRIEF-2 CRI subscale (*r* = −.30, *p* = .04).

##### ADHD symptoms

Significant negative correlations were observed bet ween right-SFG and ADHD subscales from the Conners-3 parent rating scale: right-SFG and Inattention subscale (*r* = −.35, *p* = .01) and right-SFG and Hyperactive subscale (*r* = −.37, *p* = .008).

#### Linear regression

**Figure 3.**
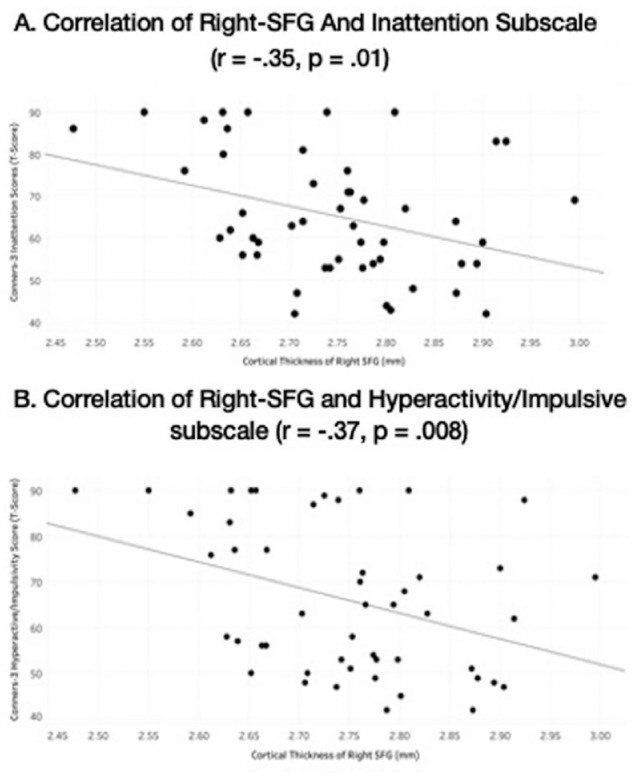
Correlations of ADHD symptom severity from Conners-3 rating scale with cortical thickness of the right superior frontal gyrus (right-SFG) across all subjects, right-SFG and inattention subscale (*r* = −.35, *p* = .01), right-SFG and hyperactive subscale (*r* = −.37, *p* = .008).

Linear regression models indicated that a significant amount of the variance in the Conners-3 Inattentive (*F* (1, 48) = 6.83, *p* = .012, *R*^2^ = .125) and Conners-3 Hyperactive (*F* (1,48) = 7.54, *p* = .008, *R*^2^ = .136) subscales were explained by right-SFG cortical thickness for the combined sample including both the ADHD and TDC groups (see Figure 3). This suggests that 12.5% of the variance in inattention and 13.6% of the variance in hyperactivity/impulsivity were accounted for by right-SFG cortical thickness. Linear regressions were also completed with Parent ratings of EF, the BRIEF-2 CRI, and predicted a significant amount of variance *F* (1, 48) = 4.57, *p* = .038, *R*^2^ = .09). There were no significant regression models for different EF assessments or any of the other EF parent rating scales. Age, biological sex, and IQ measures were not included in the model as there were no correlations observed between these measures with right-SFG cortical thickness.

## Discussion

The current study aimed to investigate neuroanatomical biomarkers and its associated clinical significance in children with ADHD. The results showed thinner right-SFG in children with ADHD, but not in the ACC. We did not observe a significant group difference in overall EF performance. However, some challenges with perseverative errors were observed on the CPT-III task. Significant EF challenges were reported by parents of children of ADHD. The present study also showed significant relationships between right-SFG cortical thickness and ADHD inattentive, ADHD hyperactive symptoms and Parent ratings of EF (BRIEF-2 CRI subscale), indicating possible clinical relevance of right-SFG in ADHD development and presentation.

Consistent with our hypothesis and replicating previous study from our lab (Yang et al., 2015) this study found significantly reduced cortical thickness in the right-SFG in the ADHD sample. Cortical thinning, specifically in the frontal regions, is associated with the delayed maturation hypothesis (Almeida et al., 2010; McLaughlin et al., 2014; Shaw et al., 2007; Stanley et al., 2008; Yang et al., 2015). Generally, regions around the Prefrontal Cortex (PFC), including right-SFG, develop slower than other areas of the brain, with peak thickness expected at approximately age 7.5 in typically developing children (Shaw et al., 2007). Shaw et al. (2007) reported that children with ADHD reach peak cortical thickness in the PFC much later, at about 10.5 years of age. This delay in maturation could explain the behavioral and cognitive symptoms observed by parents and teachers, as the PFC is involved in attention, emotional regulation, and higher-level functions, including goal management, working memory, and inhibition (Franke et al., 2018; Shaw et al., 2006). The PFC receives projections from the dopaminergic pathways through subcortical regions (Volkow et al., 2004, 2011) and makes connections with other neural networks, such as the frontoparietal and frontotemporal regions (Zillmer et al., 2008). Furthermore, studies have also shown that subregions of the SFG (part of the PFC) are involved in distinct cognitive process with the posterior part of the SFG, including the supplementary motor area (SMA) mainly activated by motor tasks, the lateral part of the SFG is involved in execution within working memory and the medial part being part of the default mode network (Li et al., 2013). While the current study did not specifically investigate the anatomical differences of these subregions, it would be important to further investigate these in future studies. Overall, the finding of thinner right-SFG in children with ADHD has important behavioral and cognitive implications and supports the neurobiological underpinning of ADHD.

The present study did not find any significant difference in cortical thickness in the ACC. Previous research within this domain has been inconsistent, with only one study finding significant differences in the ADHD group (Bledsoe et al., 2013), while other studies did not find significant differences (Hoogman et al., 2019; Yang et al., 2015). The difference in our results could be attributed to methodological differences and ADHD samples. For example, Bledsoe et al. (2013) used Query, Design, Estimate, and Contrast (QDEC) for group analysis, which may result in different findings than using SPSS. The current study followed analysis protocols set up by the ENIGMA consortium, which did not include using the QDEC method (Hoogman et al., 2019). The Bledsoe et al. (2013) ADHD sample also included only ADHD Predominantly Combined presentations, while the current study included all three subtypes. It is possible that there are differences in cortical maturation between the three presentations of ADHD, an area that needs further research and clarification. Lastly, the current study used DSM-5 diagnostic criteria to classify ADHD individuals, while the Bledsoe et al. (2013) study used DSM-4 classification. It is likely possible that the differences in diagnostic criteria could have impacted the selection of the ADHD samples and subsequent results.

Executive Function challenges are commonly observed in children with ADHD (Willcutt et al., 2005). The current study found significant EF difficulties based on parent reports. However, EF challenges were observed only on the perseverative errors on the CPT-3 measure in our participants with ADHD. Perseverative errors are described as the inappropriate repetition of a previous response in place of the current target. These errors could indicate challenges with the ability to inhibit a previous response (Fischer-Baum & Rapp, 2012). Perseverative errors could also be indicative of impulsivity (Conners, 2014). As presented in Table 4, the result from our study further supports the correlations observed between CPT-3 perseverative errors and parent ratings on the Conners-3 Hyperactive/Impulsivity subscale, suggesting that individuals with more hyperactive/impulsive symptoms are likely to make more perseverative errors. Regardless of the mechanisms associated with perseverative errors, the results from the current study suggest that children with ADHD face difficulties with perseverative errors. This finding is supported by a previous study with Brazilian students, where individuals with ADHD made more perseverative errors compared to age-matched control participants (Miranda et al., 2012). Overall, the results from the study indicate the need to use both parent ratings of EF and performance-based measure to understand EF difficulties in children with ADHD. These additional measures could help design targeted strategies and applied interventions that parents and teachers can provide to help alleviate some of the challenges faced by individuals with ADHD.

It is also essential to acknowledge that some children with ADHD perform well in the lab setting, where they get one-on-one attention. Research studies often include rewards that may be motivating for individuals with ADHD. Other studies have reported differences between parent ratings of EF skills compared to performance in lab-based settings (Toplak et al., 2009). Given that children in the current ADHD participant group had similar IQ scores compared to the typically developing peers, it is possible that they have learned skills that help manage their EF deficits. Studies in the literature have reported that reading ability and IQ can impact EF performances (Kofler et al., 2019). Another potential bias may be the self-selection of study participants. It is possible that individuals with severe ADHD and more EF difficulties do not consider participating in research studies that are time-consuming. Future studies with more deliberate outreach to reach wider participants are required. Overall, the findings from the present study are important as they show the need to measure EF using a variety of EF measurements.

One of the novel findings from this study is the significant negative correlations of ADHD symptoms (both inattention and hyperactivity) and parent ratings of EF with right-SFG cortical thickness. Regression models further showed that right-SFG cortical thickness predicted 12.5% of the variance in inattentive symptoms, 13.6% of the variance in hyperactive symptoms, and 9% of the Cognitive Regulation EF challenges. These results could suggest a possible brain-behavior link between cortical thickness and ADHD core symptomology. Previous studies have indicated that reduced cortical thickness can be associated with worse clinical outcomes in ADHD and other childhood disorders (Shaw et al., 2006; Sowell et al., 2008). Functional neuroimaging studies have shown that abnormal activation in the frontal regions in children with ADHD (Cortese et al., 2014; Rubia, 2018) suggests a possible association with clinical outcomes. These correlational findings are important as they have the potential to demonstrate the importance of right-SFG maturation. One of the criticisms of the DSM-5 diagnostic criteria is the classification of symptoms in a categorical fashion when symptoms of inattention and hyperactivity/impulsivity are dimensional and lie along a continuum (Dalgleish et al., 2020; Kraemer, 2007). These results could facilitate more larger scale research studies to explore right-SFG cortical thickness in other neurodevelopmental disorders. Given the recent findings between the overlap of ADHD symptoms and EF difficulties in ASD and obsessive-compulsive disorder (OCD; Kushki et al., 2019), future studies are encouraged to investigate the overlap of neuroanatomical correlates in different disorders to better understand common etiologies.

The present study did not find any correlations between right-SFG and EF task performances. While there were no a priori hypotheses about directional relationships, these findings are unexpected given the functionality of the frontal regions in EF tasks. It is possible that this study included a small sample size that was not able to detect EF differences. Moreover, we can also speculate that while there may be structural changes in specific regions, these structural changes do not necessarily indicate functional differences. The brain-behavior dichotomy has been a long-standing issue and one that requires further evaluation and replication by future studies.

Overall, this study provides promising findings, but the results should be understood with limitations. The cross-sectional design of the current study limits the predictability of long-term outcomes associated with thinner right-SFG in our ADHD sample. Future longitudinal research is required to better understand the long-term impacts of delayed maturation of right-SFG in the ADHD population. The existent study sample size may be considered small as we were unable to study differences between the three ADHD presentation subtypes, gender or biological sex. As such, future studies with a larger sample size are needed to replicate the current findings. Additionally, ADHD participants were referred by clinicians and were not necessarily medication naïve. It is possible that early interventions including long-term medication use and behavioral strategies may have enabled the participants in the current study to perform somewhat better than what is generally expected.

In summary, the study’s novelty is the expansion of previous findings showing thinner right-SFG in children with ADHD with clinical relevance using a dimensional approach to ADHD core symptoms of hyperactivity/impulsivity and inattention and parent ratings of EF. The second important finding from our results is that while brain-related challenges may be observed in the neuroimaging data, it does not often translate into cognitive and behavioral difficulties as measured through performance-based tasks. The study results emphasize the need for continued research using multimodal tools (both performance based EF tasks and informant ratings) to investigate biomarkers of ADHD. With the rising prevalence rates of ADHD and the need for early intervention, these biomarkers will allow for early detection and subsequently provide targeted interventions for pediatric ADHD patients.
